# eHealth Practices in Cancer Survivors With BMI in Overweight or Obese Categories: Latent Class Analysis Study

**DOI:** 10.2196/24137

**Published:** 2020-12-03

**Authors:** Annie Wen Lin, Sharon H Baik, David Aaby, Leslie Tello, Twila Linville, Nabil Alshurafa, Bonnie Spring

**Affiliations:** 1 Department of Nutrition Benedictine University Lisle, IL United States; 2 Department of Preventive Medicine Northwestern University Chicago, IL United States; 3 Department of Supportive Care Medicine City of Hope Medical Cancer Center Duarte, CA United States; 4 Department of Medical Social Sciences Northwestern University Chicago, IL United States

**Keywords:** eHealth, patient communication, cancer survivorship, obesity, behavior

## Abstract

**Background:**

eHealth technologies have been found to facilitate health-promoting practices among cancer survivors with BMI in overweight or obese categories; however, little is known about their engagement with eHealth to promote weight management and facilitate patient-clinician communication.

**Objective:**

The objective of this study was to determine whether eHealth use was associated with sociodemographic characteristics, as well as medical history and experiences (ie, patient-related factors) among cancer survivors with BMI in overweight or obese categories.

**Methods:**

Data were analyzed from a nationally representative cross-sectional survey (National Cancer Institute’s Health Information National Trends Survey). Latent class analysis was used to derive distinct classes among cancer survivors based on sociodemographic characteristics, medical attributes, and medical experiences. Logistic regression was used to examine whether class membership was associated with different eHealth practices.

**Results:**

Three distinct classes of cancer survivors with BMI in overweight or obese categories emerged: younger with no comorbidities, younger with comorbidities, and older with comorbidities. Compared to the other classes, the younger with comorbidities class had the highest probability of identifying as female (73%) and Hispanic (46%) and feeling that clinicians did not address their concerns (75%). The older with comorbidities class was 6.5 times more likely than the younger with comorbidities class to share eHealth data with a clinician (odds ratio [OR] 6.53, 95% CI 1.08-39.43). In contrast, the younger with no comorbidities class had a higher likelihood of using a computer to look for health information (OR 1.93, 95% CI 1.10-3.38), using an electronic device to track progress toward a health-related goal (OR 2.02, 95% CI 1.08-3.79), and using the internet to watch health-related YouTube videos (OR 2.70, 95% CI 1.52-4.81) than the older with comorbidities class.

**Conclusions:**

Class membership was associated with different patterns of eHealth engagement, indicating the importance of tailored digital strategies for delivering effective care. Future eHealth weight loss interventions should investigate strategies to engage younger cancer survivors with comorbidities and address racial and ethnic disparities in eHealth use.

## Introduction

More than 17 million cancer survivors reside in the United States, and simulation models predict that the survivorship population will increase to 22 million by January 2030 [1]. The growing prevalence of cancer survivors represents a significant health care challenge especially since they have higher risk for treatment-related morbidities (eg, cardiovascular disease) and cancer than the general population [2-4]. Obesity is considered a major risk factor for chronic disease, modifiable with energy-restricted, high-quality diets, and consistent physical activity [5]. Yet the prevalence of obesity continues to increase rapidly among cancer survivors [6] despite medical recommendations to maintain a healthy weight [7]. The prevalence of obesity among adult cancer survivors has increased 10% since 1997, a significantly faster rate than among those without a history of cancer [6]. However, several issues restrict cancer survivors from accessing nutrition services, including inadequate reimbursement coverage, providers’ heavy clinical load, and providers’ limited nutrition or behavior change training [8,9]. To increase access to nutrition care, digital technology support for weight management and health promotion (eHealth) is being developed to facilitate healthy lifestyle change [10-12] and patient-clinician communication [13,14].

Many eHealth interventions for cancer survivors, delivered through smartphone apps and internet websites, promote a high-quality diet and physical activity through behavior change techniques [15], such as goal-setting [16-21], self-monitoring of behavior [16-27], modeling of behavior [28], and behavioral feedback [16-19,21,24,26,28,29]. eHealth interventions have shown some promise for assisting cancer survivors with health-promoting behavior change and weight loss [30,31] yet the one size fits all approach is unlikely to be effective for this population [32]. Individuals in the increasingly culturally and linguistically diverse survivor population may have different medical experiences, as well as different digital access and engagement [33]. The few studies [34-37] that have investigated associations among eHealth use, sociodemographic characteristics, and medical history examined the general population rather than cancer survivors. In these studies [34-37], researchers found poorer engagement in eHealth practices among adults who are older, male, in a lower annual income bracket, less healthy, or without a regular provider. Even less is known about how different care experiences are associated with different types of eHealth practices. However, a recent study [38] found that negative medical experiences (ie, low perceived patient-centeredness) were associated with greater engagement in self-management eHealth practices only among those with less education and not among those with more education, suggesting that eHealth use can vary as a function of sociodemographic factors and medical experiences. Further investigation is warranted to understand how and why different combinations of these factors are associated with varied eHealth practices among cancer survivors with BMI in overweight or obese categories. Latent class analysis is a statistical approach that allows an investigation of how the intersection of several patient-related factors are associated with eHealth use. This type of analysis is useful when there are several variables that can contribute to heterogeneity, such as that observed among cancer survivors and can facilitate understanding to guide optimization of eHealth promotion among different underlying cancer survivor subgroups.

The primary objective of the study was to determine whether distinct classes can be identified based on sociodemographic characteristics, medical history, and medical experiences (eg, patient-related factors) of cancer survivors with BMI in overweight or obese categories. We also investigated whether class membership was associated with eHealth practices for weight management and patient-clinician communication among cancer survivors with BMI in overweight or obese categories.

## Methods

### National Cancer Institute Health Information National Trends Survey

National Cancer Institute’s Health Information National Trends Survey (NCI HINTS) is an ongoing cross-sectional data collection program for nationally representative data about health- and cancer-related communication in the United States. Details regarding the NCI HINTS sampling framework have been previously published [39]. During 2017-2018, self-administered questionnaires from NCI HINTS 5 Cycles 1 and 2 were mailed to households (address-based sampling). Surveys were deemed ineligible if ≤49% of the first 2 sections of the questionnaire were completed. The NCI HINTS 5 Cycles 1 and 2 comprised 6862 participants who returned their questionnaires to investigators, with a final collective response rate of 25%. Of these questionnaires, 6789 (99%) were considered completed by study investigators. In our study, participant data were excluded if respondents did not have a cancer history (n=3735) and had a BMI <25 kg/m^2^ (n=2324).

### Variables

All variables were categorical and were collected in NCI HINTS 5 Cycles 1 and 2. Sociodemographic variables included age, gender, race/ethnicity, and education level. Also included in the analysis were degree of weight above a healthy weight (overweight, class I obesity; class II obesity; class III obesity [40]), presence of medical conditions (diabetes, cardiovascular disease, or depression; hypertension; arthritis), frequency of medical visits in the past year, quality of care, health insurance status, access to medical records, and access to a regular provider. Medical experience characteristics included whether patients felt that (1) their feelings and emotions were addressed by clinicians, (2) they were involved in medical decisions, (3) their clinicians made certain that they understood next steps of care, (4) they received clear explanations from their clinicians, and (5) they were confident in their ability to take care of their own health. Response options for each medical experience questionnaire item were dichotomous (yes or no).

Nine eHealth items (outcome variables) were available across both cycles: access to a health app (1 item); use of electronic means to seek personal medical information (2 items), use of tablets or smartphones to track health and facilitate medical discussions (4 items), and use of the internet as a health resource (2 items). Response options for these items were dichotomous (yes or no). Since deidentified data were available for public use from the National Cancer Institute, ethical approval was not required for this secondary data analysis.

### Statistical Analysis

All analyses were conducted using STATA statistical software (version 15; StataCorp LLC). We used latent class analysis to empirically identify classes for cancer survivors with BMI in overweight or obese categories who exhibited similar sociodemographic and psychosocial characteristics [41]. The latent class model included sampling weights to account for the study design and to generate estimates and make inferences that reflect the population. The number of classes was selected using the Akaike information criterion (AIC); Schwarz Bayesian information criterion (BIC); Rissanen sample-size adjusted BIC; entropy, with higher values indicating better classification of individuals; and ease of interpretation (ie, the classes distinguished differences from a practical perspective). We examined a series of models, progressing from a 1-class model to a 10-class model, and compared the models using AIC, adjusted BIC, and entropy descriptive fit indices (Table 1) to identify the optimal number of classes [42,43].

**Table 1:** Latent class model selection diagnostics.

Classes, n	G^2^ deviance statistic	AIC^a^	BIC^b^	Adjusted^c^ BIC	Entropy
1	7815.99	7879.99	8026.96	7925.35	1.00
2	7424.36	7554.36	7852.91	7646.52	0.56
3	7074.80	7270.80	7720.92	7409.73	0.74
4	6930.33	7192.33	7794.02	7378.05	0.77
5	6845.61	7173.61	7926.87	7406.12	0.74
6	6648.00	7042.00	7946.83	7321.30	0.77
7	6574.32	7034.32	8090.72	7360.40	0.82
8	6447.51	6973.51	8181.48	7346.37	0.82
9	6300.65	6892.65	8252.19	7312.29	0.83
10	6238.49	6896.49	8407.60	7362.92	0.83

^a^AIC: Akaike information criterion.

^b^BIC: Bayesian information criterion.

^c^Rissanen sample size adjustment.

We determined that the 3-class model was optimal (AIC 7270.80; adjusted BIC 7409.73; entropy 0.74). Specifically, all indicators of model fit (decreased AIC and adjusted BIC, higher entropy) revealed the 2-class model fit better than the 1-class model, and the 3-class model fit better than the 2-class model. Although the slightly lower AIC and adjusted BIC values, and slightly higher entropy indicated the 4-class model fit better than the 3-class model, the 3-class model demonstrated both (1) a relatively larger decrease in the AIC and adjusted BIC values (2-class to 3-class compared to 3-class to 4-class) and (2) and a similar entropy (0.74 in 3-class vs 0.77 in 4-class). Also, the 4-class model seemed to separate Class 1 from the 3-class model into 2 distinct classes; however, these classes did not differ in any meaningful or interpretable way. The 3-class model provided the most clinically interpretable groups.

Maximum conditional probabilities for the categorical indicator variables (ie, sociodemographic, medical, and psychosocial factors) were used to characterize each class. Variables with probabilities greater than 0.50 were highly endorsed [44]. We used logistic regression to examine whether latent class membership was associated with different eHealth behaviors. Each eHealth behavior was modeled separately, using the latent classes as predictors in the model. We evaluated differences between classes using the pseudo class method, with 20 imputations. The pseudo-class method [45] provides conservative estimates of standard error and perform optimally for models with moderate entropy (0.60) and competitively for models with large entropy (0.80). Logistic regression analyses did not adjust for covariates since classes were derived from sociodemographic factors, medical history, and medical experiences, and thus their covariance was already incorporated into the analysis. We present odds ratios (ORs) and 95% confidence intervals from the logistic regression models.

## Results

### Sample Characteristics

The sample of cancer survivors with BMI in overweight or obese categories (N=730) had a mean age of 66.8 (SD 11.9) years, and these participants were mostly non-Hispanic White individuals (499/730, 76.3%) (Table 2). There was a slightly higher proportion of females (396/730, 55.1%) than males (323/730, 44.9%). Most had a BMI considered overweight (383/730, 52.5%), had health insurance (694/730, 97.3%), and a regular health care provider (624/730, 86.4%). Approximately half of the participants had been offered online access to medical records (313/730, 51.4%). Overall, for all 3 classes, participants had nearly equal probability of being offered online access to medical records (range 43%-54%).

**Table 2:** Demographic characteristics of the sample (N=730).

Characteristic		Value, n (%)
**Age**		
	Less than 49 years	58 (8.1)
	50-64 years	226 (31.7)
	65-74 years	250 (35.1)
	75 years or older	178 (25.0)
**Gender**		
	Male	323 (44.9)
	Female	396 (55.1)
**Race/ethnicity**		
	Non-Hispanic White	499 (76.3)
	Black or African American	78 (11.9)
	Hispanic	54 (8.3)
	Hawaiian/Pacific Islander, Alaskan Native, Asian, or Multiracial^a^	23 (3.5)
**Education**		
	High School or less	202 (28.2)
	Some college, professional school	239 (33.4)
	College graduate	275 (38.4)
**BMI category [40]**		
	Overweight	383 (52.5)
	Obese, class I	214 (29.3)
	Obese, class II	75 (10.3)
	Obese, class III	58 (7.9)
**Diabetes, heart condition, or depression**		
	Present	405 (57.0)
	Absent	306 (43.0)
**Hypertension**		
	Present	449 (62.3)
	Absent	272 (37.7)
**Arthritis**		
	Present	351 (48.5)
	Absent	373 (51.5)
**How many times did you go to a health professional (doctor, nurse) for care**		
	None	39 (5.4)
	1-3 times	292 (40.6)
	4+ times	388 (54.0)
**Quality of care**		
	Excellent	300 (44.8)
	Very good	231 (34.5)
	Good	112 (16.7)
	Fair	23 (3.4)
	Poor	3 (0.4)
**Health insurance**		
	Yes	694 (97.3)
	No	19 (2.7)
**Offered online access to your medical records**		
	Yes	313 (51.4)
	No	296 (48.6)
**Confidence in own ability to take care of health**		
	Completely confident	7 (1.0)
	Very confident	30 (4.1)
	Somewhat confident	189 (26.1)
	A little confident	345 (47.7)
	Not confident at all	153 (21.1)
**Regular provider**		
	Yes	624 (86.4)
	No	98 (13.6)
**Feelings addressed**		
	Yes	642 (95.8)
	No	28 (4.2)
**Involved in decisions**		
	Yes	660 (97.9)
	No	14 (2.1)
**Understood next steps**		
	Yes	666 (99.1)
	No	6 (0.9)
**Explained clearly**		
	Yes	668 (99.3)
	No	5 (0.7)

^a^These data were grouped for statistical analysis (due to the very small number of participants and model fit).

Multimedia Appendix 1 shows the percentage of participants within each class and the resulting conditional response probabilities of endorsing items, given class membership.

### Classes

Class 1 accounted for 41% of the population (Multimedia Appendix 1). The majority of class 1 was less than 65 years old (77%), had higher than high school education level (80%), and identified as being non-Hispanic White individuals (80%). In this class, there was a higher probability of having a BMI in overweight and obese class I categories (91%) and a lower probability of having medical conditions—diabetes, cardiovascular disease, or depression; hypertension; arthritis—than in the other classes (range 9%-31%). Members in class 1 predominantly had health insurance (98%), visited a regular provider (82%), and felt little to somewhat confident in their ability to take care of their own health (73%). Most reported having positive interactions with their clinicians: they believed that their feelings were addressed (95%), felt involved in decisions (100%), understood next steps in care (100%), and felt that health-related topics were clearly explained (100%). Class 1 was subsequently labeled *younger with no comorbidities*.

Class 2 represented the smallest class accounting for 4% of the population. A slight majority of its members were less than 64 years old (57%; Multimedia Appendix 1). Compared to the other classes, class 2 had the highest probability of identifying as female (73%) and having a high school education level or less (60%). The probability of class members identifying as Black or Hispanic adults (63%) was substantially higher than in classes 1 (17%) and 3 (16%). Class 2 had the highest probability of having a BMI in obese class II and III categories (63%) and having medical conditions (% range: 48-95%), and probabilities for this class of seeking care from a health care professional (31%), having a regular provider (43%), and having health insurance (24%) were lower than for other classes. Class 2 had a higher probability of reporting low quality of care (37%) than the other classes; they were more likely to believe their feelings were not addressed by health care professionals (75%) and to feel uninvolved in decisions (73%). Yet there was a high probability of feeling—at a minimum—very confident in their ability to take care of their own health (56%). Class 2 was subsequently labeled *younger with comorbidities*.

Class 3 represented the largest class and accounted for 55% of the population (Multimedia Appendix 1). The majority of class 3 was 65 years old or above (71%), identified as non-Hispanic White individuals (83%), and had a BMI within either overweight or obese class I categories (81%). There was an even distribution regarding education level among its members. Members of class 3 predominantly had health insurance (99%), had a regular provider (90%), expressed feeling a little to somewhat confident in their ability to take care of health (77%), and reported positive interactions with their clinicians, similar to class 1. Specifically, members in class 3 felt that in medical care, their feelings were addressed (97%), they were involved in decisions (99%), understood the next steps in care (100%), and felt that things were explained clearly (100%). There were differences in medical outcomes between classes 1 and 3, with class 3 having higher probabilities of being diagnosed with all comorbidities—diabetes, cardiovascular disease, or depression; hypertension; arthritis except for obesity (range 64%-80% vs 9%-31%). Class 3 was subsequently labeled *older with comorbidities*.

### Association of eHealth Behaviors and Latent Classes

Table 3 presents the associations of eHealth behaviors with latent classes. Logistic regression analyses indicated that, compared with the younger with comorbidities class, the older with comorbidities class had more than a 6-fold increase in the odds of sharing health information from an electronic device or smartphone with a health professional (OR 6.53, 95% CI 1.08-39.43). There were no significant differences in the likelihood of engaging in eHealth behaviors between younger with no comorbidities and younger with comorbidities classes (Table 3). The younger with no comorbidities class had greater odds than the older with comorbidities class of engaging in self-management eHealth practices that do not involve a health care provider, including using a computer to look for health information (OR 1.93, 95% CI 1.10-3.38), using a tablet or smartphone to track progress toward a health-related goal (OR 2.02, 95% CI 1.08-3.79), and using the internet to watch health-related videos on YouTube (OR 2.70, 95% CI 1.52-4.81) (Table 3).

**Table 3:** Logistic regression models predicting eHealth behaviors using latent classes as predictors.

eHealth Behaviors	Younger with no comorbidities vs younger with comorbidities^a^		Younger with no comorbidities vs older with comorbidities^a^		Older with comorbidities vs younger with comorbidities^a^	
	Odds ratio	95% CI	Odds ratio	95% CI	Odds ratio	95% CI
On your tablet or smartphone, do you have any apps related to health and wellness?	1.28	(0.29, 5.58)	1.61	(0.87, 2.96)	0.80	(0.18, 3.63)
In the past 12 months have you used a computer, smart phone, or other electronic means to look for health or medical information for yourself?	2.73	(0.73, 10.14)	1.93	(1.10, 3.38)	1.41	(0.39, 5.11)
In the past 12 months have you used a computer, smart phone, or other electronic means to look up medical test results?	2.16	(0.55, 8.48)	1.63	(0.99, 2.67)	1.33	(0.34, 5.14)
Has your tablet or smartphone helped you track progress on a health-related goal, such as quitting smoking, losing weight, or increasing physical activity?	2.28	(0.47, 11.02)	2.02	(1.08, 3.79)	1.13	(0.23, 5.46)
Has your tablet or smartphone helped you make a decision about how to treat an illness or condition?	1.25	(0.24, 6.42)	1.16	(0.63, 2.15)	1.08	(0.20, 5.71)
Has your tablet or smartphone helped you in discussions with your health care provider?	1.33	(0.22, 7.85)	0.67	(0.37, 1.23)	1.97	(0.31, 12.50)
Have you shared health information from either an electronic monitoring device or smartphone with a health professional within the last 12 months?	3.63	(0.57, 23.22)	0.56	(0.27, 1.13)	6.53	(1.08, 39.43)
In the last 12 months, have you used the internet to participate in an online forum or support group for people with a similar health or medical issue?	2.11	(0.12, 37.70)	2.50	(0.68, 9.16)	1.40	(0.12, 16.19)
In the last 12 months, have you used the internet to watch a health-related video on YouTube?	1.84	(0.42, 8.11)	2.70	(1.52, 4.81)	0.68	(0.15, 2.99)

^a^This class was used as the reference.

## Discussion

Despite the substantial investment in advancing eHealth to extend patient care [46], there is insufficient evidence about how sociodemographic factors, medical history, and medical experiences affect how different groups of cancer survivors use eHealth. As obesity is both prevalent and a significant risk factor for future multimorbidity among cancer survivors, our study objective was to characterize patterns of eHealth use among distinct classes of cancer survivors with BMI in overweight or obese categories. Three classes emerged: younger with no comorbidities; younger with comorbidities; older with comorbidities. People in the older with comorbidities class were less likely to use eHealth self-management technologies than those in the younger-no comorbidities class. However, when compared to those in the younger with comorbidities class, people in the older with comorbidities class were more likely to share health information from an eHealth device with a health professional.

Among cancer survivors with comorbidities, older adults were more likely than younger adults to share their eHealth data with a health care provider in order to facilitate patient-clinician communication. Our finding supports the supposition that eHealth is a promising tool to facilitate patient-clinician communication for older cancer survivors with comorbidities. In comparison, those in the younger with comorbidities class were less likely to have a regular provider, have health insurance, feel involved in medical decisions, or feel they understood next steps of care. They were also more likely to identify as Black or Hispanic individuals and have a lower education level. The characteristics observed in the younger with comorbidities class were consistent with previous reports that Black and Hispanic participants receive less health care than non-Hispanic White participants, and that cancer survivors with lower education are less likely to discuss health-promoting behaviors [47-49]. We also observed that the younger with comorbidities class did not emerge within the 2-class model. This observation suggests that minority groups among cancer survivors with BMI in overweight or obese categories can easily go unnoticed and underrepresented in health care despite having different medical experiences and being at increased risk of having a medical condition, relative to non-Hispanic White adults. A valuable opportunity exists for clinicians and researchers to identify strategies that will improve the medical experiences of underserved minority groups, while leveraging eHealth technology to facilitate health-promoting behaviors.

Compared to those in the older with comorbidities class, cancer survivors in the younger with no comorbidities class were more likely to use a computer to research health information, use a tablet or smartphone to track progress on a health-related goal, and watch health-related videos on YouTube—all types of self-management eHealth behaviors. These differences seemed to be largely driven by the combination of age and medical history as the 2 classes shared similar characteristics for other sociodemographic factors and medical experiences. However, those in the older with comorbidities and younger with no comorbidities classes showed no differences for other eHealth behaviors, such as (1) having health-related apps on their devices, (2) accessing health records for test results, (3) using electronic devices to treat a condition with clinicians, and (4) participating in a health-related support group. Collectively, these results demonstrate that although younger age and better health status jointly predict greater engagement in using eHealth for self-management, there is no generational divide in having health-related apps, accessing electronic health records, and sharing eHealth data with clinicians among cancer survivors with BMI in overweight or obese categories. Our results show agreement with mixed evidence that age is associated with eHealth use [34,35,37], and echo findings indicating that better health was associated with greater eHealth use to track health and goals [34,35].

The strengths of this study include the use of a large nationwide sample drawn from NCI HINTS which allowed us to use weightings to generate nationally representative estimates. Although the sample analyzed for the current study comprised less than 5% of the NCI HINTS study sample, the estimates are reflective of the population of cancer survivors with BMI in overweight or obese categories. Despite several eHealth weight management interventions in survivor populations, this is the first study to investigate how eHealth is used to manage health and relate to health care providers [50]. An additional strength was the ability to investigate different forms of eHealth usage separately, rather than in aggregate, which allowed us to identify who was more likely to use specific eHealth features to promote weight management and patient-clinician communication. A few limitations should be noted as well. We were unable to determine whether eHealth use would differ for diet, physical activity, or smoking behaviors since the NCI HINTS items did not distinguish between types of health-promoting behaviors. Another limitation is that eHealth use and cancer status were self-reported and, therefore, susceptible to recall bias. Although the data were weighted to generate nationally representative estimates, generalizability may still be limited by reliance on participant self-selection. Replication is warranted using different nationally representative study samples with further investigation on environmental factors, such as rural-urban differences [51]. Additionally, the temporal relationship between patient-related factors and eHealth use has yet to be established.

There is growing interest in the development and usability of eHealth to guide health-promoting behaviors for cancer survivors [52-54], particularly as there is limited access to nutrition services at cancer centers [9]. This study provides new evidence about the feasibility and usability of eHealth among cancer survivors with BMI in overweight or obese categories by investigating how sociodemographic factors, medical history, and medical experiences co-vary with eHealth behaviors. While our results suggest that all cancer survivors use eHealth, some groups engage with eHealth technologies in different ways. Thus, this study highlights the importance of considering the eHealth needs and usage patterns of different types of cancer survivors when developing digital interventions to support health promotion and patient-clinician communication. Our study also reveals that race/ethnicity, as well as medical attributes and experiences, predict eHealth use—lending support to the idea that sociodemographic, medical history, and clinician interactions can collectively influence eHealth engagement. Further efforts to develop eHealth recommendations tailored for different groups of cancer survivors are needed to optimize survivors’ ability to use digital tools to promote health behaviors and reduce treatment-related morbidities and obesity.

## Appendix

Multimedia Appendix 1 Latent class model based on patient-related factors of cancer survivors with BMI in overweight or obese categories.

## Abbreviations

AIC: Akaike information criterion
BIC: Bayesian information criterion
NCI HINTS: National Cancer Institute Health Information National Trends Survey
OR: odds ratio
