# Supplementary material for: eHealth Practices in Cancer Survivors With BMI in Overweight or Obese Categories: Latent Class Analysis Study
Source: JMIR Cancer. 2020 Dec 3;6(2):e24137. doi: 10.2196/24137 (PMC7746487; doi:10.2196/24137)
Supplement: Multimedia Appendix 1 [file cancer_v6i2e24137_app1.docx]

| **Latent Class Model of Cancer Survivors with Overweight or Obesity Based on Sociodemographic Factors, Medical Conditions and Experiences^a^** | | | | |
| --- | --- | --- | --- | --- |
|  |  | **Class 1** | **Class 2** | **Class 3** |
| Assigned Label |  | Younger-no comorbidities | Younger-comorbidities | Older-comorbidities |
| % in Latent Class |  | 41.4 | 4.1 | 54.5 |
| Conditional Probability (%)^b^ |  |  |  |  |
| Age | Age <= 49 yrs | 25.4 | 24.7 | 5.6 |
|  | Age 50-64 yrs | 51.7 | 32.1 | 23.2 |
|  | Age 65-74 yrs | 16.3 | 9.2 | 38.6 |
|  | Age 75 or older | 6.6 | 34.1 | 32.6 |
| Gender | Male | 54.4 | 27.2 | 46.7 |
|  | Female | 45.6 | 72.8 | 53.3 |
| Race/Ethnicity | Non-Hispanic White | 80.1 | 32.5 | 82.5 |
|  | Black or African American | 6.7 | 16.8 | 10.8 |
|  | Hispanic | 9.8 | 46.3 | 5.4 |
|  | Other^c^ | 3.5 | 4.3 | 1.3 |
| Education | High School or less | 20.0 | 59.9 | 42.4 |
|  | Some college, professional school | 42.5 | 24.9 | 37.6 |
|  | College graduate | 37.5 | 15.2 | 20.0 |
| BMI Category | Overweight | 61.6 | 32.8 | 49.7 |
|  | Obese Class 1 | 28.9 | 4.0 | 32.0 |
|  | Obese Class 2 | 6.6 | 47.9 | 8.4 |
|  | Obese Class 3 | 2.9 | 15.2 | 9.9 |
| Medical Conditions: Diabetes, Heart Condition, or Depression | Present | 30.5 | 95.0 | 67.7 |
|  | Absent | 69.5 | 5.0 | 32.3 |
| High Blood Pressure | Present | 27.5 | 91.2 | 80.0 |
|  | Absent | 72.5 | 8.8 | 20.0 |
| Arthritis | Present | 9.3 | 48.4 | 64.0 |
|  | Absent | 90.7 | 51.6 | 36.0 |
| How many times did you go to a health professional (doctor, nurse) for care | None | 7.0 | 30.5 | 2.3 |
|  | 1-3 times | 63.3 | 40.8 | 34.5 |
|  | 4+ times | 29.7 | 28.7 | 63.2 |
| Quality of care | Excellent | 47.9 | 43.9 | 44.4 |
|  | Very good | 34.8 | 0.6 | 35.0 |
|  | Good | 15.5 | 18.6 | 19.0 |
|  | Fair | 1.8 | 26.0 | 1.6 |
|  | Poor | 0.0 | 10.9 | 0.0 |
| Health Insurance | Yes | 97.5 | 75.8 | 97.9 |
|  | No | 2.5 | 24.2 | 2.1 |
| Offered online access to your medical records | Yes | 53.7 | 42.6 | 46.7 |
|  | No | 46.3 | 57.4 | 53.3 |
| Confidence in own ability to take care of health | Completely confident | 0.0 | 23.4 | 1.4 |
|  | Very confident | 0.4 | 32.1 | 4.6 |
|  | Somewhat confident | 24.0 | 6.1 | 35.2 |
|  | A little confident | 49.3 | 29.9 | 41.8 |
|  | Not confident at all | 26.4 | 8.4 | 17.0 |
| Regular provider | Yes | 81.5 | 57.1 | 90.3 |
|  | No | 18.5 | 42.9 | 9.7 |
| Feelings addressed | Yes | 95.1 | 25.0 | 97.2 |
|  | No | 4.9 | 75.0 | 2.8 |
| Involved in decisions | Yes | 100.0 | 27.1 | 99.1 |
|  | No | 0.0 | 72.9 | 0.9 |
| Understood next steps | Yes | 100.0 | 58.5 | 100.0 |
|  | No | 0.0 | 41.5 | 0.0 |
| Explained clearly | Yes | 100.0 | 59.9 | 100.0 |
|  | No | 0.0 | 40.1 | 0.0 |

^a^ Latent class model is weighted to adjust for sampling design

^b^ The conditional probabilities for each variable sum within classes. For example, class 1 is 80.1% non-Hispanic White, 6.7% African American, 9.8% Hispanic, and 3.5% other race. Percentages may not sum to 100% due to rounding.

^c^ Other race includes Hawaiian/Pacific Islander, Alaskan Native, Asian, or multi-racial
